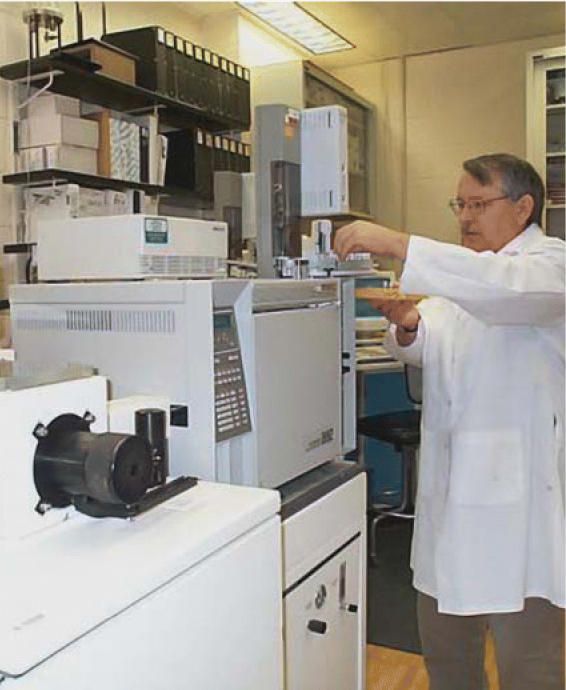# Beyond the Bench: Tracking Clues with Environmental Health Sleuths

**DOI:** 10.1289/ehp.114-a158

**Published:** 2006-03

**Authors:** Tanya Tillett

Who doesn’t love a good mystery? Add in detailed, interactive information on the latest environmental health research, and you’ve got the basic concept behind Unsolved Mysteries of Human Health: How Scientists Study Toxic Chemicals, a project developed by the Community Outreach and Education Core (COEC) of the Environmental Health Sciences Center (EHSC) and Marine and Freshwater Biomedical Sciences Center (MFBSC) at Oregon State University.

The Unsolved Mysteries website (http://www.unsolvedmysteries.oregon-state.edu/) was launched in 2005 as a supplement to provide information on the analytical instruments and techniques featured in the Hydroville Curriculum Project, a problem-based environmental health science curriculum for high school students [see “Beyond the Bench: Welcome to Hydroville!” *EHP* 112:A166 (2004)]. Since then, the Unsolved Mysteries website has grown into an independent, informative teaching tool in its own right. “Unsolved Mysteries offers a great opportunity to highlight research at the EHSC and MFBSC at Oregon State while delivering a balance between scientific accuracy and a nontechnical explanation of very complex concepts,” says COEC program coordinator Sandra Uesugi.

The core used input from EHSC investigators to develop two modules to date. Each provides information on techniques used in basic chemistry, environmental health research, and toxicology via an “unsolved mystery” based on real research scenarios.

In “The Answers Are Blowing in the Wind,” the reader meets Jenny, who hikes to the top of a mountain but finds the view blocked by haze. While atop the hazy mountain, Jenny encounters Robert, an Oregon State graduate student taking air samples. Jenny’s experience with unexpected haze at the mountaintop sets the stage for a discussion of semivolatile chemicals, their atmospheric transport across long distances to high elevations, and the use of gas chromatography–mass spectrometry to analyze trace amounts of chemicals in the air. Then students analyze the mass spectrum data from Robert’s air samples to solve the puzzle of whether the haze blew in over the water to the west or came from forest fires to the east.

In “Going With the Flow,” the reader meets Joe, who plans to catch some fish for dinner. At the fishing spot, he notices a sign warning that the fish in that area may contain elevated levels of dioxin and should be eaten only once a month. Joe wants to learn more about dioxin and its health effects, so the game warden refers him to the Oregon State EHSC. The module tells how the scientists there use flow cytometry to study immune damage. Students then compare mouse histogram data to unravel the puzzle of dioxin’s effects on the immune system.

Each module includes a “Meet Real Scientists!” section that introduces the investigators behind the research. The web-site also features virtual tours of each researcher’s lab so visitors can see examples of the tools discussed. In addition, there is a detailed glossary of technical terms and a link to additional resources.

Although the content is written for high school students, the information is also appropriate for the general public, and the site has proved to be popular not only with students but also others surfing the web. “Our inquiries have come from many diverse backgrounds including an agriculture professor in Oman teaching an instruments course, a medical school professor giving a lecture on endocrinology, a hematologist at a hospital in Cambridge, United Kingdom, a home-schooled student, and a graduate student considering a future in toxicology research,” says Uesugi.

As a testament to its value, Unsolved Mysteries received the Digital Dozen Award from the Eisenhower National Clearing-house in March 2005, an honor given to exemplary websites for educators that feature current, accurate math and/or science content, that support school improvement efforts, and that have useful multimedia features or helpful navigation. The Oregon State COEC continues to build on the website’s accomplishments by keeping the information fresh and engaging. An upcoming Unsolved Mysteries module will highlight the use of microarray techniques, focusing on zebrafish embryos as a model for understanding chemical toxicity and the impact of developmental toxicants on human health. Be ready to solve the next Unsolved Mystery in spring 2006!

## Figures and Tables

**Figure:**
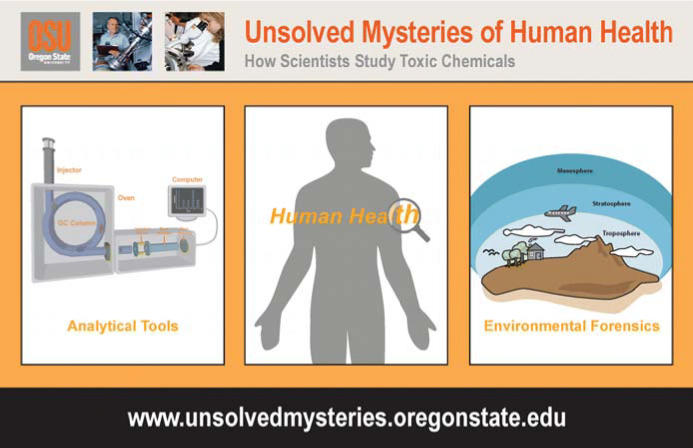
Applied tools for learning. The Unsolved Mysteries modules show students how scientists in the real world use tools like gas chromatography–mass spectrometry (right) to better understand the relationship of toxicants to our health.

**Figure:**